# *Ex vivo* susceptibilities of *Plasmodium vivax* isolates from the China-Myanmar border to antimalarial drugs and association with polymorphisms in *Pvmdr1* and *Pvcrt-o* genes

**DOI:** 10.1371/journal.pntd.0008255

**Published:** 2020-06-12

**Authors:** Jiangyan Li, Jie Zhang, Qian Li, Yue Hu, Yonghua Ruan, Zhiyong Tao, Hui Xia, Jichen Qiao, Lingwen Meng, Weilin Zeng, Cuiying Li, Xi He, Luyi Zhao, Faiza A. Siddiqui, Jun Miao, Zhaoqing Yang, Qiang Fang, Liwang Cui

**Affiliations:** 1 Department of Microbiology and Parasitology, Bengbu Medical College, Bengbu, Anhui Province, China; 2 Anhui Key Laboratory of Infection and Immunity, Bengbu Medical College, Bengbu, Anhui Province, China; 3 Department of Pathogen Biology and Immunology, Kunming Medical University, Kunming, Yunnan Province, China; 4 Xiangtan Blood Center, Xiangtan, Hunan Province, China; 5 Department of Pathology, Kunming Medical University, Kunming, Yunnan Province, China; 6 Department of Internal Medicine, Morsani College of Medicine, University of South Florida, Tampa, Florida, United States of America; Menzies School of Health Research, AUSTRALIA

## Abstract

**Background:**

Vivax malaria is an important public health problem in the Greater Mekong Subregion (GMS), including the China-Myanmar border. Previous studies have found that *Plasmodium vivax* has decreased sensitivity to antimalarial drugs in some areas of the GMS, but the sensitivity of *P*. *vivax* to antimalarial drugs is unclear in the China-Myanmar border. Here, we investigate the drug sensitivity profile and genetic variations for two drug resistance related genes in *P*. *vivax* isolates to provide baseline information for future drug studies in the China-Myanmar border.

**Methodology/Principal findings:**

A total of 64 *P*. *vivax* clinical isolates collected from the China-Myanmar border area were assessed for *ex vivo* susceptibility to eight antimalarial drugs by the schizont maturation assay. The medians of IC_50_ (half-maximum inhibitory concentrations) for chloroquine, mefloquine, pyronaridine, piperaquine, quinine, artesunate, artemether, dihydroartemisinin were 84.2 nM, 34.9 nM, 4.0 nM, 22.3 nM, 41.4 nM, 2.8 nM, 2.1 nM and 2.0 nM, respectively. Twelve *P*. *vivax* clinical isolates were found over the cut-off IC_50_ value (220 nM) for chloroquine resistance. In addition, sequence polymorphisms in *pvmdr1* (*P*. *vivax* multidrug resistance-1), *pvcrt-o* (*P*. *vivax* chloroquine resistance transporter-o), and difference in *pvmdr1* copy number were studied. Sequencing of the *pvmdr1* gene in 52 samples identified 12 amino acid substitutions, among which two (G698S and T958M) were fixed, M908L were present in 98.1% of the isolates, while Y976F and F1076L were present in 3.8% and 78.8% of the isolates, respectively. Amplification of the *pvmdr1* gene was only detected in 4.8% of the samples. Sequencing of the *pvcrt-o* in 59 parasite isolates identified a single lysine insertion at position 10 in 32.2% of the isolates. The *pvmdr1* M908L substitutions in *pvmdr1* in our samples was associated with reduced sensitivity to chloroquine, mefloquine, pyronaridine, piperaquine, quinine, artesunate and dihydroartemisinin.

**Conclusions:**

Our findings depict a drug sensitivity profile and genetic variations of the *P*. *vivax* isolates from the China-Myanmar border area, and suggest possible emergence of chloroquine resistant *P*. *vivax* isolates in the region, which demands further efforts for resistance monitoring and mechanism studies.

## Author summary

Vivax malaria is an important parasitic disease in the Greater Mekong Subregion (GMS). *Plasmodium vivax*, which is less sensitive to chloroquine, has been reported in the region. However, as part of the GMS, the drug sensitivity of *P*. *vivax* is unclear in the China-Myanmar border area. In view of the important role of antimalarial drugs in malaria elimination strategies, we investigate the drug sensitivity profile and genetic variations of the *P*. *vivax* isolates from the China-Myanmar border. We obtained baseline data of *ex vivo* sensitivities to eight antimalarial drugs and found that chloroquine-resistant *P*. *vivax* was possibly emerging this area. Studies of genetic polymorphisms in two candidate genes of drug resistance (*pvmdr1* and *pvcrt-o*) of the *P*. *vivax* isolates from this area and found association between the M908L substitution in *pvmdr1* with reduced sensitivities to and chloroquine, mefloquine, pyronaridine, piperaquine, quinine, artesunate and dihydroartemisinin. It is worth noting that the M908L substitution was almost fixed in our samples, which limits the robustness of the conclusion. Our finding suggests that more rigorous *in vivo* and *ex vivo* studies are needed to monitor the efficacy of the frontline drugs and discover drug resistance mechanisms.

## Introduction

With extensive control efforts over the last decade, the global malaria burden has been greatly reduced, which has motivated many countries to reset their goals for malaria elimination [1]. The Greater Mekong Subregion (GMS), which comprises six countries (Cambodia, China, Laos, Myanmar, Thailand, and Vietnam), is aiming to achieve the goal of regional malaria elimination by 2030 [2, 3]. Unfortunately, this plan is being challenged by the increased prevalence of *Plasmodium vivax* (*P*. *vivax*) [4], a parasite capable of surviving as a dormant form and causing relapses [5, 6]. In most vivax-endemic areas, the frontline treatment of *P*. *vivax* infections remains chloroquine/primaquine (CQ/PQ), with CQ eliminating blood-stage infections and PQ providing radical cure of the liver stages [7]. Although artemisinin‐based combination therapies (ACTs) as unified treatment for both *P*. *falciparum* and *P*. *vivax* infections in areas of co-existence of these two species may offer significant advantages [8], ACT for treating *P*. *vivax* malaria is only deployed where CQ resistance in *P*. *vivax* is evident [9]. At the China-Myanmar border, with the availability of various antimalarial drugs in the private sectors, malaria is often treated with a variety of antimalarials, which warrants monitoring of parasites’ sensitivities to these drugs.

*P*. *vivax* CQ-resistant (CQR) strains were first reported in Papua New Guinea and Indonesia [10, 11], and over the subsequent two decades, reports on CQR *P*. *vivax* parasites in other endemic countries have increased in number [7, 12]. In the GMS, although CQ remains generally effective for the treatment of *P*. *vivax* cases [13–14], there have been sporadic reports of CQR *P*. *vivax* in most GMS countries. CQR *P*. *vivax* malaria has been documented in Myanmar as early as in 1993 [15–17], and in recent years clinical failures after CQ treatment have been reported in multiple regions of Myanmar [18, 19]. Although the recurrent rates of *P*. *vivax* infections within 28 days of CQ treatment remained low in Vietnam (3.5%) and western Thailand (8%) [14, 21], CQR *P*. *vivax* isolates have been confirmed in these areas [20, 21]. Therefore, CQR *P*. *vivax* parasites have become a concern in the management of vivax malaria in the GMS, especially for achieving regional malaria elimination.

Currently, the mechanisms of CQ resistance in *P*. *vivax* remain unknown and appear to be different from those for CQR *P*. *falciparum* [7, 22]. *Pfcrt* (*P*. *falciparum CQ resistance transporter*) and *pfmdr1* (*P*. *falciparum multidrug resistance 1*) genes are frequently used to monitor *P*. *falciparum* resistance to 4-aminoquinoline drugs and mefloquine (MFQ) [23–26]. Thus, the orthologs of these two genes in *P*. *vivax*, *pvcrt-o* and *pvmdr1*, have been targeted for studying CQ resistance in *P*. *vivax*. Whereas some studies reported the association of the *pvmdr1* substitutions Y976F with CQR *P*. *vivax in vitro* [27], others did not find such an association [28–30]. Some studies have found that an increase in *pvmdr1* gene copy number was correlated with increased susceptibility to CQ, but perhaps reduced susceptibility to MFQ [31–33]. In a study from Brazil, higher expression levels of *pvcrt-o* and *pvmdr1* were shown to confer CQ resistance in *P*. *vivax* [34]. Another study found that expression levels of *pvcrt-o* did not correlate with the *ex vivo* response to CQ [35].

With wide applications of artemisinin and its derivatives in ACTs, *P*. *falciparum* resistance to artemisinin family drugs emerged, first in Cambodia [36, 37], then detected in many countries and regions in Southeast Asia [38–40]. Resistance of *P*. *falciparum* parasites was not only limited to CQ, artemisinin and its derivatives, but also to ACT partner drugs such as MFQ and piperaquine (PPQ) [23, 41, 42]. Unfortunately, with the high frequency of misdiagnosis in routine practice and the presence of mixed infections, patients with vivax malaria often found themselves treated with similar drugs for *P*. *falciparum* malaria. Thus, it would be important to determine the susceptibilities of *P*. *vivax* parasites to these drugs.

In the GMS, malaria transmission is concentrated along international borders [43]. We have been tracking the dynamics of malaria incidence along the China-Myanmar border, an area in pursuit of malaria elimination. Our studies have found that the two sides of border had very distinct malaria epidemiology [44]. Whereas malaria in Yunnan province of China has largely been eliminated, malaria in the Kachin State of Myanmar remained highly endemic, even with vivax malaria outbreaks occurring in recent years [45, 46]. Along the highly porous international border, malaria introduction into Yunnan from Myanmar becomes a major challenge for malaria elimination in China [47–49]. To address the potential emergence of CQR *P*. *vivax* in this region, we monitored clinical efficacy of the *P*. *vivax* isolates to CQ [18]. Here we attempted to use the *ex vivo* drug assay to monitor the susceptibilities of *P*. *vivax* clinical isolates to a number of commonly used antimalarial drugs. We further surveyed the polymorphisms of candidate resistance markers *pvmdr1* and *pvcrt-o* in these isolates.

## Materials and methods

### Ethical approvals

This study was approved by the Institutional Review Board of Pennsylvania State University and Kunming Medical University and written informed consent was obtained from all patients.

### Study site and parasite isolates

Eighty-five *P*. *vivax* clinical isolates were collected during the peak transmission season (June–August) in 2012–2013 and in 2015 from malaria patients (aged between 1 to 67 years) presented with acute, uncomplicated *P*. *vivax* infections at two malaria clinics located in Laiza near the China-Myanmar border (S1 Table). Malaria diagnosis was performed by microscopy and parasitemia was determined based on the number of *Plasmodium*-infected red blood cells (iRBCs) in 40,000 RBCs on Giemsa-stained thin films. Nested PCR targeting the 18S rRNA gene was subsequently performed to confirm single-species *P*. *vivax* infections [50]. Parasite staging was based on morphological characteristics described earlier [51]. Monoclonal *P.vivax* infections were confirmed using a PCR/RFLP protocol for *P*. *vivax merozoite surface protein-3α* (*pvmsp-3α*) [51]. Only patients with >0.5% parasitemia and >70% ring stage on presentation and without taking antimalarial medicine were enrolled. Three to five milliliters of blood were drawn from each patient, collected in heparin-coated tubes, stored at 37°C, and transported to a nearby laboratory for *ex vivo* drug assays within four hours of collection.

### *In vitro* drug assay

The schizont maturation assay was used to assess *ex vivo* susceptibility of *P*. *vivax* field isolates to antimalarial drugs [52]. For each isolate, 2 mL of *P*. *vivax*-infected blood were centrifuged at 1,000 rpm for 10 min, and the pellet was re-suspended in two volumes of serum-free RPMI 1640 medium. Leukocytes were removed by passing the blood through an NWF filter (Zhi Xing Bio, Bengbu, China) [53, 54]. Then, 400 μL of the packed erythrocytes were re-suspended in 19.6 mL of culture medium containing McCoy’s 5A medium (11.9 g/L, Sigma, St. Louis, MO, USA), HEPES (25 ml/L, Sigma), gentamicin (5 mg/L, Jinan Limin Pharmaceutical Co., Ltd, Jinan, China), 7.5% NaHCO3 (2.1 g/L, Sigma), 25% AB^+^ (malaria-naive donors) serum to make a 2% cell suspension. The erythrocyte suspension (100 μL/well) was dispensed into wells of Costar 96-well flat-bottom microtiter plates (Sigma) pre-dosed with antimalarial drugs.

CQ, MFQ, quinine (QN) and pyronaridine phosphate (PND) were purchased from Sigma, PPQ was obtained from Chongqing Kangle Pharmaceutical Co., Ltd (Chongqing, China), artemether (AT), dihydroartemisinin (DHA) and artesunate (AS) were obtained from Kunming Pharmaceutical Corp. (Kunming, China). The stock solutions of CQ (1279 μmol/L), QN (2560 μmol/L), and PND (4000 μmol/L) were prepared in distilled water, and PPQ (4000 μmol/L) and MFQ (640 μmol/L) in methanol. The stock solutions of AT (268mmol/L), DHA (4000 μmol/L), and AS (156 mmol/L) were made in absolute ethanol. Serial dilutions were made in 70% ethanol to obtain final concentrations of 0, 3.12, 12.5, 50, 200, 800, 3200, and 12800 nM for CQ and QN; 0, 6.25, 12.5, 25, 50, 100, 400, and 1600 nM for MFQ and 0, 4, 15.6, 62.4, 250, 500, 1000, and 4000 nM for PPQ; 0, 0.4, 1.56, 6.24, 25, 50, 100, 400 nM for DHA, AS, AT and PND. Each drug concentration was assayed in triplicate. The testing plates were dried inside a biosafety hood, sealed and stored at 4°C to be used within two weeks. After parasitized blood samples were added into the test plates, they were incubated at 37°C in a candle jar for 26–31 h depending on the stage of the parasites on admission. After 12 h of *in vitro* culture, parasite development in drug-free wells was monitored every 1–4 h. When 40% of parasites in the control wells developed into mature schizont stage, all wells were harvested to prepare thick and thin blood films [55]. Blood films were fixed with methanol, stained with 10% Giemsa (Sigma) solution for 30 min and examined microscopically under oil immersion. Schizonts with at least three well-defined chromatin dots were counted. The number of schizonts per 200 asexual stage parasites was determined for each drug concentration and normalized to that of the control well.

### Analysis of genetic polymorphisms in *pvmdr1* and *pvcrt-o* genes

Single nucleotide polymorphisms (SNPs) in *pvmdr1* and *pvcrt-o* genes were determined by PCR and sequencing. Parasite genomic DNA was extracted using the QIAmp96 DNA kit (Qiagen, Valencia, CA, USA). Primers for *pvmdr1* amplification were designed by using Primer Premier 6 (Table 1) and the primers for *pvcrt-o* were used as previously described [56]. Amplification of *pvmdr1* and *pvcrt-o* gene fragments was performed in 25 μL of volume containing 12.5 μL of 2× Phanta Max Buffer (Mg^2+^), 10 mM of each dNTP mix, 0.5 μM of each primer, 0.5 U of Super-Fidelity DNA Polymerase (Vazyme Biotech, Nanjing, China), and 1 μL of genomic DNA. The PCR cycling parameters were as follows: initial denaturation at 95°C for 3 min, followed by 35 cycles at 95°C for 15 sec, 58°C for 15 sec, 72°C for 4 min, and then final extension at 72°C for 5 min. PCR products were sequenced using the BigDye Terminator v3.1. For each sample, separate contigs were formed with the overlapping sequences in both directions using the DNASTAR program (Madison, WI, USA). Sequences were aligned with the CLUSTAL X 2.0.12 with manual editing. The sequences were aligned with the Salvador I (Sal-I) strain reference sequences retrieved from the GenBank (XM_001613678 for *pvmdr1* and AF314649 for *pvcrt-o*) to determine the presence of SNPs in the respective genes.

**Table 1:** *Ex vivo* IC_50_ values (nM) of clinical *P*. *vivax* isolates to eight antimalarial drugs.

Drugs	Total			2012–2013			2015			*P* value*
	N	Median (IQR)	Range	N	Median (IQR)	Range	N	Median (IQR)	Range	
AS	59	2.8 (1.3–4.8)	0.1–17.6	35	2.9 (1.7–4.8)	0.1–6.7	24	2.1 (0.7–5.0)	0.4–17.6	0.3883
AT	34	2.1 (1.1–3.8)	0.1–6.2	34	2.1 (1.1–3.8)	0.1–6.2	Not done			
DHA	58	2.0 (1.1–4.8)	0.2–43.6	34	2.1 (1.2–3.6)	0.2–11.5	24	1.8 (0.8–6.1)	0.4–43.6	0.9096
CQ	64	84.2 (43.1–185.2)	2.1–710.8	40	86.9 (52.1–211.0)	2.1–710.8	24	81.4 (21.0–173.4)	9.9–319.4	0.4088
MFQ	58	34.9 (25.1–52.0)	5.7–335.6	34	32.9 (25.7–45.8)	5.7–335.6	24	39.0 (21.9–56.3)	6.5–160.8	0.7694
PND	64	4.0 (2.2–10.5)	0.6–97.3	40	2.9 (1.9–4.6)	0.6–14.5	24	11.8 (6.4–64.3)	2.3–97.3	< 0.0001
PPQ	24	22.3 (11.2–109.5)	2.9–429.8	Not done			24	22.3 (11.2–109.5)	2.9–429.8	
QN	24	41.4 (18.0–95.5)	6.1–203.7	Not done			24	41.4 (18.0–95.5)	6.1–203.7	

* Comparison between isolates collected in 2012–2013 and 2015 (Mann-Whitney test).

IQR–interquartile range

### Quantification of *pvmdr1* gene copy number by real-time PCR

To estimate the copy number of *pvmdr1*, two reference plasmids containing one copy of the *pvmdr1* and *pvaldolase* genes, respectively, were generated and used as positive controls for real-time PCR analysis of *pvmdr1* copy number. Real-time PCR was carried out in 20 μL containing 1 μL of DNA, 1 μM of each primer, 10 μL of FastSart Essential DNA Green Master Mix using the following conditions: 50°C for 2 min, 95°C for 10 min, then 39 cycles at 95°C for 10 sec and 60°C for 30 sec. Threshold cycle (Ct) values were used for relative quantitation of *pvmdr1* copy number as described [57]. Copy number was determined by rounding to the nearest integer.

### Statistical analysis

Statistical analysis was performed using Graphpad Prism 6.0 (GraphPad Software, Inc, San Diego, CA, USA) and SPSS version 21.0 (SPSS Inc., Chicago, IL, USA). Ex vivo 50% inhibitory concentrations (IC_50s_) of the drugs of the clinical isolates were calculated by fitting the data to a sigmoid curve. Differences in *in vitro* drug susceptibility between groups were determined using non-parametric Mann-Whitney test. Correlation between two drugs was evaluated via the Spearman’s correlation test.

## Results

### *Ex vivo* sensitivities of parasite isolates to antimalarial drugs

Sixty-four isolates showed development to mature schizonts in the control wells and could be evaluated for drug susceptibility. Numbers of isolates that were successfully assayed were 64, 58, 64, 59, 58 and 34 for CQ, MFQ, PND, AS, DHA, and AT, respectively (Table 1). Only 24 samples were assayed for CQ, MFQ, PND, AS, DHA, QN, and PPQ, respectively in 2015. Overall, the median *ex vivo* CQ IC_50_ for the 64 *P*. *vivax* isolates was 84.2 nM (range 2.1–710.8 nM), with 18.8% (12/64) isolates having IC_50_ values above 220 nM. For the 58 isolates successfully assayed for MFQ, the median *ex vivo* IC_50_ was 34.9 nM (range 5.7–335.6 nM). For PND, the median *ex vivo* IC_50_ was 4.0 nM (range 0.6–97.3 nM). For the artemisinin derivatives, the median IC_50_ were relatively low (1.1–3.8 nM). For the 24 parasite isolates collected in 2015, the *ex vivo* IC_50_ values for PPQ was 22.3 (range 2.9–429.8 nM). Parasites had a median *ex vivo* IC_50_ value of 41.4 nM (range 6.1–203.7 nM) to QN (S1 Fig, S2 Table).

**Fig 1:**
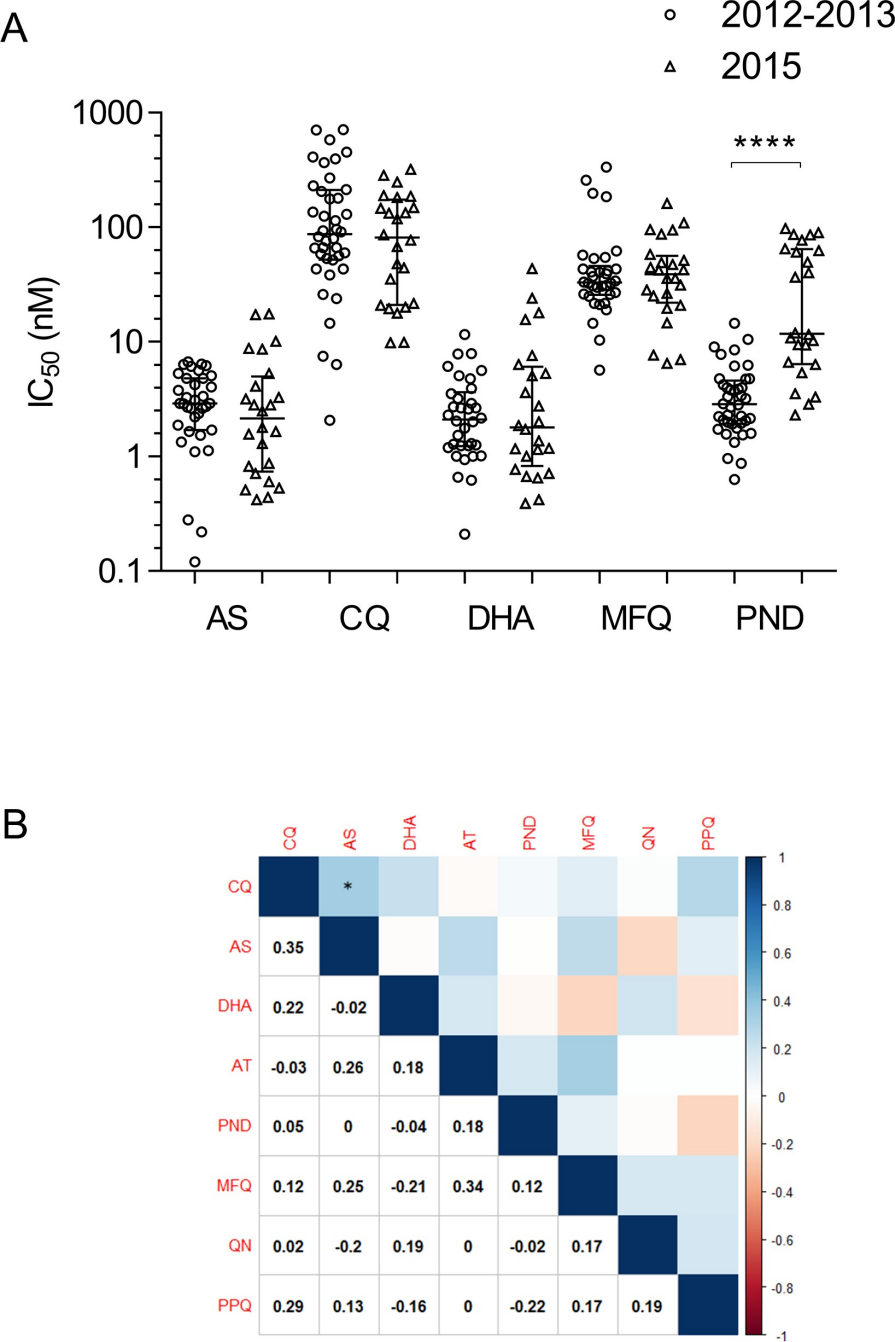
IC_50_ values of *P*. *vivax* isolates for each antimalarial drug. **A. Comparison of *ex vivo* IC**_**50**_ **values (in nM) to five antimalarial drugs between parasite isolates collected in 2012–2013 and 2015.** **** indicates *P* < 0.0001 (Mann-Whitney U test). **B. Spearman’s correlation analysis between IC**_**50**_ **values of parasite isolates to eight antimalarial drugs.** The values of the correlation coefficient were colored based on color scale shown on the right. * indicates significant correlation at *P* < 0.05. AS, artesunate; AT, artemether; CQ, chloroquine; DHA, dihydroartemisinin; MFQ, mefloquine; PND, pyronaridine; PPQ, piperaquine; QN, quinine.

We also compared susceptibilities between parasite isolates collected in 2012–2013 and 2015 to five drugs. For AS, DHA, CQ, and MFQ, the differences in IC_50_ values between the two study periods were not significant (*P* > 0.05, Table 1, Fig 1A). For PND, there was a significant increase in IC_50_ in parasites collected in 2015 (*P* < 0.0001, Table 1, Fig 1A). In order to detect whether sensitivities to different drugs were correlated, Spearman’s correlation analysis was performed between IC_50s_ of pairs of drugs. There is no correlation between IC_50_ values of parasite isolates to eight antimalarial drugs in the study, expect a weak correlation between AS and CQ (Fig 1B, r = 0.35, *P* < 0.05).

### Polymorphisms in *pvmdr1* and *pvcrt-o* genes

The *pvmdr1* gene was successfully sequenced in 52 *P*. *vivax* isolates. Compared with the Sal-I sequence, 12 non-synonymous substitutions (T409M, S513R, G520D, G698S, L845F, A861E, M908L, T958M, Y976F, F1076L, K1393N, and S1450L) and 8 synonymous substitutions (K44K, L493L, G501G, T529T, K555K, T1348T, S1358S and E1396E) were identified (Table 2). Two of these substitutions G698S and T958M were fixed in the parasite populations. M908L was present in 98.1% of the parasite populations. Two substitutions Y976F and F1076L, proposed to be associated with CQ resistance [57], were detected in the parasite population with drastically different prevalence: Y976F was rare (3.8%), whereas F1076L was highly prevalent (78.8%). Furthermore, a new substitution K1393N was identified with more than 20% prevalence in the clinical samples analyzed. When all the mutations in the two study periods were compared, the prevalence of F1076L increased from 67.9% in 2012–2013 to 91.7% in 2015, and the difference was significant (*P* = 0.0463).

**Table 2:** The prevalence of *Pvmdr1* substitutions in 52 parasite isolates.

	Prevalence of substitutions [no. (%)]			
Mutations	2012–2013 (n = 28)	2015 (n = 24)	Total (n = 52)	*P* value*
K44K^#^	11 (39.3)	9 (37.5)	20 (38.5)	1.0000
T409**M**	4 (14.3)	6 (25.0)	10 (19.2)	0.4829
L493L^#^	2 (7.1)	1 (4.2)	3 (5.8)	1.0000
G501G^#^	0	2 (8.3)	2 (3.8)	0.2081
S513**R**	6 (21.4)	1 (4.2)	7 (13.5)	0.1072
G520**D**	2 (7.1)	5 (20.8)	7 (13.5)	0.2273
T529T^#^	25 (89.3)	23 (95.8)	48 (92.3)	0.6146
K555K^#^	0	2 (8.3)	2 (3.8)	0.2081
G698**S**	28 (100)	24 (100)	52 (100)	-
L845**F**	1 (3.6)	1 (4.2)	2 (3.8)	1.0000
A861**E**	5 (17.9)	8 (33.3)	13 (25.0)	0.2201
M908**L**	28 (100)	23 (95.8)	51 (98.1)	0.4615
T958**M**	28 (100)	24 (100)	52 (100)	-
Y976**F**	2 (7.1)	0	2 (3.8)	0.4932
F1076**L**	19 (67.9)	22 (91.7)	41 (78.8)	0.0463^*^
T1348T^#^	1 (3.6)	5 (20.8)	6 (11.5)	0.0836
S1358S^#^	1 (3.6)	2 (8.3)	3 (5.8)	0.5895
K1393**N**	8 (28.6)	6 (25.0)	14 (26.9)	1.0000
E1396E^#^	4 (14.3)	0	4 (7.7)	0.1149
S1450**L**	4 (14.3)	5 (20.8)	9 (17.3)	0.7161

* Comparison between isolates between 2012–2013 and 2015 (Fisher’s exact test).

^#^ Synonymous substitutions (K44: AAG→AAA, L493L: TTA→CTA, G501G: GGT→GGG, T529T: ACA→ACG, K555K: AAG→AAA, T1348T: ACC→ACT, S1358S: TCC→TCT, S1358S: TCC→TCT, E1396E: GAG→GAA)

A total of 16 haplotypes (409/513/520/698/845/861/908/958/976/1076/1393/1450) were generated (Table 3). The most prevalent haplotype TSG**S**LA**LM**Y**L**KS (25%) carried 4 substitutions, which was followed by **M**SG**S**LA**LM**Y**LN**S (15.4%) carrying 6 substitutions. More than 50% parasite isolates carried 4 and 5 substitutions, in the *pvmdr1* gene.

**Table 3:** Prevalence of *Pvmdr1* haplotypes in clinical *P*. *vivax* isolates.

Substitution	Haplotype*	Prevalence [N (%)]
Triple	TSG**S**LA**LM**YFKS	3 (5.8)
Quadruple	T**R**G**S**LA**LM**YFKS	2 (3.8)
	TSG**SF**A**LM**YFKS	1 (1.9)
	TSG**S**LA**LM**Y**L**KS	13 (25)
Quintuple	**M**SG**S**LAM**M**Y**LN**S	1 (1.9)
	**M**SG**S**LA**LM**Y**L**KS	1 (1.9)
	T**R**G**S**LA**LM**YF**N**S	4 (7.7)
	TSG**SF**A**LM**Y**L**KS	1 (1.9)
	TSG**S**L**ELM**Y**L**KS	5 (9.6)
	TSG**S**L**ELM**YF**N**S	1 (1.9)
	TSG**S**LA**LMFL**KS	1 (1.9)
	TSG**S**LA**LM**Y**L**K**L**	3 (5.8)
Sextuple	**M**SG**S**LA**LM**Y**LN**S	8 (15.4)
	T**R**G**S**LA**LMFL**KS	1 (1.9)
	TS**DS**L**ELM**Y**L**KS	1 (1.9)
Septuple	TS**DS**L**ELM**Y**L**K**L**	6 (11.5)

* Substitutions at amino acids 409/513/520/698/845/861/908/958/976/1076/1393**/**1450.

Given the potential contribution of *pvmdr1* copy number variation to altered susceptibility to CQ and MFQ [31–33], the copy number of the *pvmdr1* gene was determined by real-time PCR. For the 62 samples where *pvmdr1* copy number was successfully determined, 95.2% of the parasite isolates had one copy, whereas 3 (4.8%) isolates had two copies of the *pvmdr1* gene.

The *pvcrt-o* gene was successfully sequenced in 59 *P*. *vivax* isolates. Compared with the Sal -I sequence, no SNPs were identified. However, 19 (32.2%) isolates carried a K10 insertion (AAG codon) in exon I of the *pvcrt-o* gene.

### Correlation between polymorphisms and *ex vivo* drug susceptibilities

**Fig 2:**
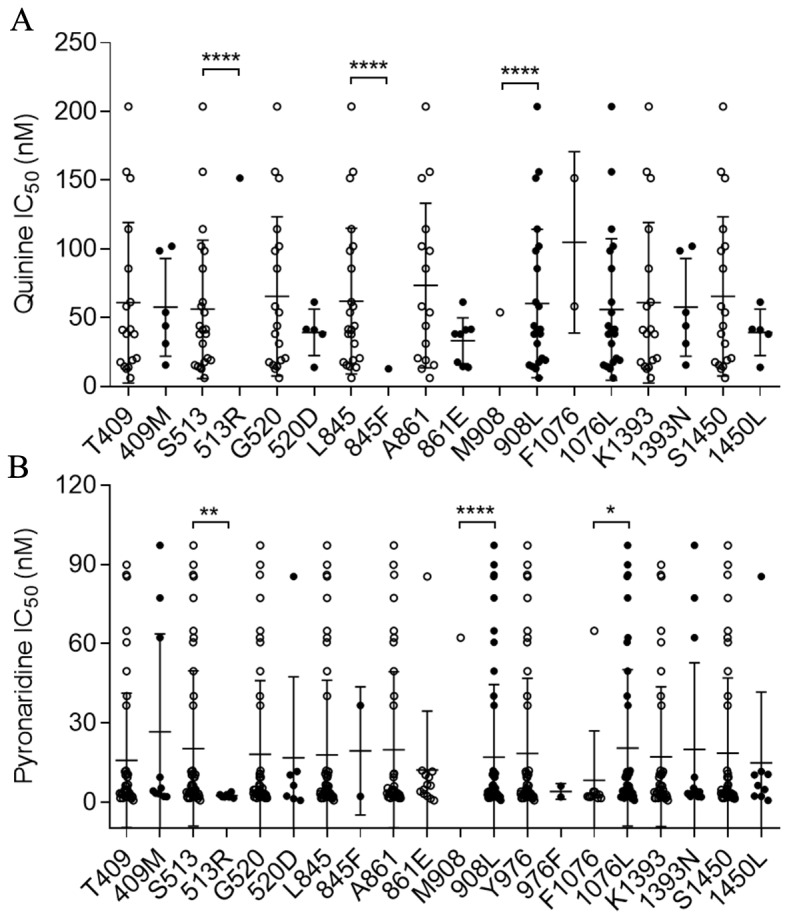
**Association of SNPs in *pvmdr1* with *ex vivo* susceptibilities to quinine (A) and pyronaridine (B).** *, **, and **** indicate significant differences between the two alleles at *P* < 0.05, < 0.01 and <0.0001, respectively.

Of the twelve non-synonymous *pvmdr1* SNPs (T409M, S513R, G520D, G698S, L845F, A861E, M908L, T958M, Y976F, F1076L, K1393N, and S1450L), only M908L had a significant association with the IC_50_ to CQ (P < 0.05; S2 Fig). Based on the cut-off IC_50_ value of 220 nM used by others [27], 12 *P*. *vivax* isolates were categorized as CQR and 52 isolates as CQ-sensitive. S513R, L845F, and M908L have a significant association with the IC_50_ to QN (*P* < 0.01; Fig 2). S513R, M908L, and F1076L had a significant association with the IC_50_ to PND (*P* < 0.01; Fig 2). In addition, the M908L substitutions was also association with decreased sensitivities to CQ, PPQ, AS, DHA, and MFQ, (*P* < 0.05, S2 Fig). Comparison of the prevalence of the seven *pvmdr1* substitutions between these two CQ sensitivity categories did not reveal any significant differences (*P* > 0.05, Table 4). With regard to *pvmdr1* copy number variation, increased *pvmdr1* copy number did not appear to significantly alter parasites’ susceptibilities to most drugs tested except MFQ. A single parasite isolate with more than one copy of *pvmdr1* was associated with a significant decrease in IC_50_ to MFQ (S3 Fig).

**Table 4:** Comparison of *pvmdr1* and *pvcrt-o* substitutions between chloroquine sensitive and -resistant isolates.

Gene	Amino acid substitutions	% of CQ-resistant*	% of CQ- susceptive*	*P value*
*Pvmdr1*	T409**M**	12.5	20.5	>0.05
	S513**R**	25.0	11.4	>0.05
	G520**D**	25.0	11.4	>0.05
	L845**F**	0	4.5	>0.05
	A861**E**	50.0	20.5	>0.05
	M908**L**	100	97.7	>0.05
	Y976**F**	0	4.5	>0.05
	F1076**L**	75.0	79.5	>0.05
	K1393**N**	37.5	25.0	>0.05
	S1450**L**	25.0	15.9	>0.05
*Pvcrt-o*	K10 insertion	36.4	31.3	>0.05

*Using 220 nM as the cutoff IC_50_ for chloroquine (CQ) resistance. ≥ 220 nM was considered as CQ resistant isolates, and < 220 nM were considered as CQ-sensitive isolates.

Analysis of the *pvcrt-o* gene revealed no significant association of the K10 insertion with reduced susceptibility to drugs tested (*P* > 0.05; S4 Fig). In addition, the prevalence of *pvcrt-o* K10 insertion in CQ-sensitive and CQ-resistant isolates was also similar (*P* > 0.05; Table 4).

## Discussion

Rational use of antimalarial drugs is an important factor in the combat against malaria. CQ has remained as the first-line treatment of *P*. *vivax* malaria in most vivax-endemic regions for more than 60 years [58, 59]. This extensive use of CQ has likely exerted selective pressure on *P*. *vivax* parasites resulting in the evolution CQ resistance in many endemic areas, including the GMS [4, 16, 20, 60–63]. In addition, treatment of falciparum malaria with ACTs may have inevitably led to changes in drug responses in *P*. *vivax* parasites due to the occurrence of mixed species as well as misdiagnosed infections. Here, we examined the *ex vivo* susceptibilities of *P*. *vivax* field isolates collected from the China-Myanmar border area to several commonly used antimalarials. For all drugs tested, the ranges of responses of the parasite isolates were wide, suggesting the existence of parasites with much reduced sensitivities to the commonly used antimalarials. However, for the majority of drugs tested, we did not find significant correlations in responses, suggesting that these variations may have different genetic basis.

Recent efficacy studies suggested the emergence of CQ resistance in this area of the GMS [18, 20, 64]. In comparison with other *ex vivo* assays performed in endemic areas such as central China [58], Thailand [65], South Korea [66], and Colombia [67], and Indonesia [35], the CQ IC_50_ value for the China-Myanmar border isolates was greater than almost all the IC_50_ values for parasites from other regions with the exception of Thailand and Indonesia. Since the cutoff for defining CQ resistance in *P*. *vivax* is lacking, some researchers used 220 nM, whereas others used 100 nM as the cutoff IC_50_ value for CQ resistance [27, 68]. Using 220 nM as the cutoff value, we found that about 18.8% (12/64) of *P*. *vivax* isolates from the China-Myanmar border could be considered resistant or having reduced sensitivity to CQ. This result aligned well with our earlier report of diminished sensitivity of *P*. *vivax* parasites to CQ in the study area [18]. Unfortunately, the lack of the clinical efficacy data for the parasite isolates used in this *ex vivo* study did not allow us to make further comparison to derive a more realistic resistance cutoff value for parasites from this area. Nonetheless, the results from *ex vivo* and clinical efficacy studies collectively demonstrate a need for continued monitoring of the efficacy of the frontline treatment CQ for *P*. *vivax* malaria during the elimination phase.

We also measured *ex vivo* susceptibilities of the parasite isolates to other antimalarials and the general findings are that for most of the drugs the IC_50_ values span a wide range, and the median IC_50_s did not differ significantly between the two study periods. However, several drugs are worthy to mention. Compared with other regions, the MFQ IC_50_ of *P*. *vivax* is generally lower at the China-Myanmar border, which may be related to the fact that MFQ has not been used as a major drug to treat malaria in China or Myanmar. In comparison, a formulation for intravenous injection of PND has been widely used in this region, often administered together with other antimalarials for faster relief of clinical symptoms. This might underlie the significant increase in IC_50_ for parasite isolates collected in 2015 as compared to those collected in 2012–13. We also did not find significant correlations in drug sensitivity among quinoline drugs and artemisinin family drugs, suggesting that in the emergence of CQ resistance in *P*. *vivax*, ACT could be considered as an option for the treatment of CQ-resistant vivax malaria like the practice in Indonesia [8, 9]. Of note, sensitivities to artemisinin family drugs may also need to be measured using an assay similar to the *in vitro* ring-stage survival assay developed for *P*. *falciparum* [69, 70].

No molecular markers are confirmed for CQ resistance in *P*. *vivax*, while *pvmdr1* and *pvcrt-o* have often been queried since these two genes are involved in CQ resistance in *P*. *falciparum* [12]. Sequencing of full-length *pvmdr1* in 52 *P*. *vivax* isolates identified 12 non-synonymous substitutions compared to the Sal-I sequence, among which two substitutions (G698S and T958M) are fixed, indicating divergence of the GMS parasite population from the South American parasite populations. It is noteworthy that the M908L and T958M substitutions were shown to be associated with reduced *ex vivo* CQ susceptibility [71]. We found that M908L substitution in our samples was associated with reduced sensitivity to CQ, PPQ, AS, DHA, QN, PND and MFQ. Whereas Y976F was present at a low frequency, F1076L occurred at a very high frequency. This finding is consistent with other parasite populations from Myanmar, where Y976F was also rare [64]. However, the pattern is not similar to those from other border regions such as the Thai-Cambodian border where the Y976F incidence is high [72].

Increased *pvmdr1* copy number was associated with reduced sensitivity to MFQ in some studies [31–33, 73]. Like in *P*. *falciparum* [74], *pvmdr1* amplification was also rare at the China-Myanmar border and only detected in three isolates (3/64). Statistical analysis showed an increase in IC_50_ to MFQ in one isolate with 2 *pvmdr1* copies, but the result is not robust given the single parasite involved.

Analysis of the *pcvrt-o* gene revealed an occurrence of the AAG insertion in the exon I of the gene, leading to an extra amino acid at position 10 (termed as “K10 insertion”). This insertion was also identified in other parasite populations. In our studied parasites, K10 was present in 32.2% parasites, which is comparable to other parts of the GMS such as Cambodia (46%) and Myanmar (56%) [66], but it is much higher than India (5.6%) [75]. Although earlier studies reported a significant increase in IC_50_ to CQ in parasites with the K10 insertion [27], we only found its association with slightly reduced susceptibility to AS. Similarly, this insertion was present in almost equal proportions in arbitrarily categorized CQ-resistant and -sensitive parasites based on the 220 nM cutoff.

*Ex vivo* drug assays conducted on *P*. *vivax* parasites from the China-Myanmar border to eight antimalarial drugs did not show significant changes in susceptibility between the two study periods, with the exception of a significant increase in IC_50_ of PND from 2012–13 to 2015. Although the *in vitro* limit of CQ resistance has not been clarified, we believe that with 18.8% of the parasite isolates having IC_50_ values above the 220 nM cutoff, CQ resistance has emerged in this area. Sequencing of two candidate genes of drug resistance identified 12 amino acid substitutions in *pvmdr1*, but only one amino acid insertion in *pvcrt-o*. The *pvmdr1* M908L substitution in *pvmdr1* in our samples was associated with reduced sensitivity to CQ, PPQ, AS, DHA, QN, PND and MFQ. In the face of emergence of CQ resistance in this region, continuous *in vivo* and *ex vivo* studies are needed to monitor the efficacy of the frontline drugs and discover the drug resistance mechanisms.

## Supporting information

S1 Table Characteristics of *P*. *vivax* patients. (DOCX)

S2 Table IC_50_ values to eight antimalarial drugs and haplotypes of *P*. *vivax* isolates. (DOCX)

S1 Fig Heat map of *ex vivo* IC_50_ values to eight antimalarial drugs. (TIF)

S2 Fig Association between *pvmdr1* substitutions and IC_50_ values to six antimalarial drugs. ****, *P* < 0.001.
(TIF)

S3 Fig Comparison of IC_50_ values for six antimalarials between parasites with single copy and multicopies of *pvmdr1* gene. ****, *P < 0*.*0001*, Mann Whitney U test.
(TIF)

S4 Fig Association between *pvcrt-o* K10 insertion and IC_50_ values to six antimalarial drugs. (TIF)
