# Supplementary material for: Ex vivo susceptibilities of Plasmodium vivax isolates from the China-Myanmar border to antimalarial drugs and association with polymorphisms in Pvmdr1 and Pvcrt-o genes
Source: PLoS Negl Trop Dis. 2020 Jun 12;14(6):e0008255. doi: 10.1371/journal.pntd.0008255 (PMC7314094; doi:10.1371/journal.pntd.0008255)
Supplement: S1 Table — (DOCX) [file pntd.0008255.s001.docx]

**S1 Table** Characteristics of *P. vivax* patients.

| Patient feature | Value |
| --- | --- |
| Total no. of patients (% male) | 64 (56.25) |
| Median age (year) (rang) | 18 (1-67) |
| Mean temp (°C) (rang) | 38.10 (37-40) |
| Occupation |  |
| Indoor worker ^a^ | 12 (18.75%) |
| Manual labor ^b^ | 5 (7.81) |
| Farmer | 8 (12.5%) |
| Business person | 0 |
| Soldier | 9 (14.06%) |
| Student | 21 (32.81%) |
| Other | 9 (14.06%) |
| Malaria history ^c^ |  |
| Yes | 1 (1.56%) |
| No | 63 (98.44%) |
| The density of the parasite ^d^ (mean ± STD [rang]) | 0.76±0.23 (0.51-1.17) |
| Ring density (mean ± STD [rang]) | 83.84±8.8 (70.21-96.58%) |

^a^ Indoor workers include office workers, housewives/housekeepers, and teachers.

^b^ Manual labor include factory/construction workers, lumberjack, plantation workers, temporary job/labor, hunters, miners, herdsman and gardeners/bush clearing.

^c^ Malaria history reported in the previous 12 months.

^d^ Protozoan density was the number of infected red blood cells in 40,000 red blood cells.
