# Supplementary material for: Ex vivo susceptibilities of Plasmodium vivax isolates from the China-Myanmar border to antimalarial drugs and association with polymorphisms in Pvmdr1 and Pvcrt-o genes
Source: PLoS Negl Trop Dis. 2020 Jun 12;14(6):e0008255. doi: 10.1371/journal.pntd.0008255 (PMC7314094; doi:10.1371/journal.pntd.0008255)
Supplement: S2 Table — (DOCX) [file pntd.0008255.s002.docx]

**S2 Table** IC50 values to eight antimalarial drugs and haplotypes of *P. vivax* isolates.

| Sample | CQ  (nM) | AS (nM) | DHA (nM) | AT (nM) | PND (nM) | MFQ (nM) | QN  (nM) | PPQ (nM) | Haplotypes* |
| --- | --- | --- | --- | --- | --- | --- | --- | --- | --- |
| N2.013.6.10.283 | 114.10 | 3.10 | 3.21 | 4.45 | 0.63 | 30.58 | - | - | TS**DS**L**ELM**Y**L**K**L** |
| N2.013.6.7.268 | 43.10 | 2.91 | 0.94 | 2.79 | 3.97 | 30.85 | - | - | T**R**G**S**LA**LM**YF**N**S |
| N2.013.6.11.296 | 6.32 | 0.22 | 0.21 | 2.99 | 1.92 | 26.15 | - | - | T**R**G**S**LA**LM**YF**N**S |
| N2.646 | 366.20 | 4.03 | 5.61 | 2.05 | 0.96 | 37.15 | - | - | - |
| N2.650 | 95.85 | 2.22 | 2.64 | 3.02 | 4.24 | 24.97 | - | - | **M**SG**S**LA**LM**Y**LN**S |
| N2.659 | 82.52 | 2.73 | 2.30 | 2.24 | 1.33 | 40.48 | - | - | TS**DS**L**ELM**Y**L**KS |
| LZCH.1359 | 38.28 | 5.31 | 2.90 | 3.88 | 6.14 | 29.99 | - | - | TSG**S**LA**LMFL**KS |
| N2.638 | 25.93 | 1.10 | 1.53 | 1.02 | 0.87 | 21.59 | - | - | - |
| N2.985 | 581.70 | 2.68 | 2.17 | 1.93 | 2.24 | 14.5 | - | - | **M**SG**S**LA**LM**Y**LN**S |
| LZCH.12.7.1151 | 14.48 | 0.12 | 1.24 | 0.10 | 2.23 | 43.13 | - | - | TSG**SF**A**LM**YFKS |
| LZCH.12.7.1215 | 58.18 | 1.34 | 1.19 | 1.11 | 1.94 | 62.15 | - | - | T**R**G**S**LA**LMFL**KS |
| N2.6.1.4 | 65.93 | 2.91 | 2.64 | 2.26 | 3.71 | 33.99 | - | - | - |
| LZCH.12.8.1430 | 56.34 | 6.21 | 1.00 | 6.21 | 2.29 | 39.33 | - | - | TSG**S**LA**LM**YFKS |
| N3.146 | 410.30 | 6.39 | 6.13 | 1.46 | 4.77 | 25.97 | - | - | TSG**S**L**ELM**Y**L**KS |
| N2.1411 | 710.80 | 6.13 | 11.54 | 4.26 | 7.80 | 197.9 | - | - | - |
| N2.830 | 66.26 | 2.62 | 2.04 | 2.45 | 3.40 | 32.56 | - | - | TSG**S**LA**LM**Y**L**KS |
| N2.808 | 268.10 | 4.81 | 3.93 | 4.17 | 2.05 | 257.4 | - | - | T**R**G**S**LA**LM**YF**N**S |
| N2.809 | 79.47 | 1.53 | 1.28 | 1.05 | 1.99 | 10.37 | - | - | TSG**S**LA**LM**Y**L**KS |
| N3.139 | 129.60 | 3.35 | 3.52 | 4.47 | 3.19 | 53.47 | - | - | TSG**S**L**ELM**Y**L**KS |
| N3.129 | 176.30 | 1.65 | 1.24 | 1.80 | 10.54 | - | - | - | TSG**S**LA**LM**Y**L**K**L** |
| N3.260 | 51.61 | 5.06 | 1.30 | 4.20 | 8.48 | 185.2 | - | - | - |
| N2.751 | 212.80 | 4.22 | 5.08 | 1.42 | 2.14 | 40.83 | - | - | TSG**S**LA**LM**Y**L**K**L** |
| LZCH.2638 | 91.34 | 1.87 | 1.26 | 1.40 | 14.46 | - | - | - | - |
| N2.013.6.12.309 | 23.88 | 2.62 | 0.66 | 0.79 | 1.77 | 54.7 | - | - | - |
| N3.96 | 135.30 | 3.28 | 0.62 | 2.24 | 4.76 | - | - | - | TSG**S**LA**LM**Y**L**K**L** |
| N2.606 | 124.90 | 4.80 | 2.01 | 0.77 | 2.08 | 18.98 | - | - | **M**SG**S**LA**LM**Y**L**KS |
| LZCH.2478 | 93.36 | 2.37 | 1.76 | 1.00 | 3.33 | 5.68 | - | - | **M**SG**S**LA**LM**Y**LN**S |
| LZCH.12.6.961 | 205.60 | 5.64 | 1.00 | 1.15 | 1.54 | 57.04 | - | - | TSG**S**LA**LM**YFKS |
| N2.630 | 394.40 | 3.77 | 7.84 | 0.87 | 2.68 | 21.2 | - | - | - |
| LZCH.12.6.840 | 59.59 | 1.70 | 2.60 | 0.92 | 3.98 | 33.21 | - | - | TSG**S**L**ELM**YF**N**S |
| LZCH.12.6.912 | 453.00 | 6.34 | 2.72 | 1.53 | 2.82 | - | - | - | TSG**S**LA**LM**Y**L**KS |
| LZCH.N36.2012.00286 | 2.06 | 0.28 | 7.87 | 3.79 | 9.06 | 26.83 | - | - | - |
| LZCH12.7.1025 | 43.08 | 1.12 | 1.01 | 3.89 | 1.59 | 43.29 | - | - | T**R**G**S**LA**LM**YFKS |
| LZCH.12.7.1081 | 66.11 | 6.69 | 4.76 | 2.07 | 1.56 | - | - | - | TSG**S**LA**LM**Y**L**KS |
| N2.1455 | 179.60 | 2.88 | - | - | 6.27 | - | - | - | - |
| MSY30-5 | 53.38 | - | - | - | 3.80 | 335.55 | - | - | TSG**S**LA**LM**Y**L**KS |
| N2.426 | 75.95 | - | - | - | 4.72 | 21.65 | - | - | TSG**S**LA**LM**Y**L**KS |
| N2.444 | 229.00 | - | - | - | 3.87 | 32.46 | - | - | - |
| CMH.499 | 7.51 | - | - | - | 1.73 | 41.66 | - | - | - |
| N3.232 | 705.10 | - | - | - | 2.88 | 31.13 | - | - | T**R**G**S**LA**LM**YF**N**S |
| LZCH-1319 | 9.91 | 1.65 | 5.28 | - | 40.18 | 44.70 | 114.55 | 13.35 | TSG**S**LA**LM**Y**L**KS |
| LZCH-1336 | 76.95 | 0.87 | 1.37 | - | 60.55 | 28.30 | 6.05 | 11.67 | TSG**S**LA**LM**Y**L**KS |
| LZCH-1339 | 188.60 | 0.42 | 5.08 | - | 2.86 | 25.13 | 151.48 | 17.95 | T**R**G**S**LA**LM**YFKS |
| LZCH-1370 | 20.80 | 2.82 | 43.62 | - | 3.54 | 14.66 | 85.83 | 7.68 | TSG**S**LA**LM**Y**L**KS |
| LZCH-1475 | 319.40 | 0.44 | 7.62 | - | 11.93 | 6.48 | 17.65 | 26.70 | TSG**S**L**ELM**Y**L**KS |
| LZCH-1480 | 148.60 | 1.30 | 1.17 | - | 89.95 | 86.97 | 203.74 | 16.46 | TSG**S**LA**LM**Y**L**KS |
| LZCH-1483 | 146.20 | 17.26 | 0.77 | - | 6.62 | 95.13 | 14.46 | 184.40 | TSG**S**L**ELM**Y**L**KS |
| LZCH-1516 | 284.00 | 8.65 | 1.18 | - | 85.5 | 160.80 | 38.03 | 17.17 | TS**DS**L**ELM**Y**L**K**L** |
| LZCH-1573 | 133.40 | 4.09 | 2.76 | - | 36.6 | 31.24 | 12.87 | 11.36 | TSG**SF**A**LM**Y**L**KS |
| LZCH-1594 | 44.27 | 1.79 | 1.15 | - | 3.29 | 35.85 | 98.71 | 86.20 | **M**SG**S**LA**LM**Y**LN**S |
| LZCH-1641 | 85.78 | 0.71 | 0.42 | - | 9.47 | 51.52 | 38.32 | 117.30 | TSG**S**L**ELM**Y**L**KS |
| LZCH-1665 | 48.03 | 2.50 | 3.60 | - | 64.9 | 26.54 | 58.19 | 369.20 | TSG**S**LA**LM**YFKS |
| LZCH-2029 | 21.65 | 3.17 | 0.39 | - | 9.4 | 35.90 | 101.95 | 199.80 | **M**SG**S**LA**LM**Y**LN**S |
| LZCH-2049 | 118.60 | 10.11 | 2.00 | - | 10.3 | 19.58 | 61.34 | 84.93 | TS**DS**L**ELM**Y**L**K**L** |
| LZCH-2090 | 17.75 | 0.60 | 1.86 | - | 5.37 | 20.85 | 31.24 | 30.32 | **M**SG**S**LA**LM**Y**LN**S |
| LZCH-2366 | 132.50 | 5.30 | 0.71 | - | 77.33 | 94.50 | 44.16 | 5.87 | **M**SG**S**LA**LM**Y**LN**S |
| LZCH-2374 | 67.74 | 1.57 | 1.73 | - | 62.3 | 109.30 | 53.85 | 11.19 | **M**SG**S**LAM**M**Y**LN**S |
| LZCH-2586 | 186.40 | 17.57 | 0.65 | - | 49.6 | 42.63 | 19.15 | 429.80 | TSG**S**LA**LM**Y**L**KS |
| NB-42 | 9.85 | 3.27 | 15.66 | - | 10.8 | 7.68 | 20.62 | 7.43 | TSG**S**LA**LM**Y**L**KS |
| NB-43 | 181.60 | 0.53 | 6.35 | - | 86.1 | 47.12 | 156.09 | 73.64 | TSG**S**LA**LM**Y**L**KS |
| NB-45 | 20.05 | 0.51 | 1.00 | - | 6.32 | 7.01 | 13.89 | 2.87 | TS**DS**L**ELM**Y**L**K**L** |
| NB-48 | 19.50 | 8.75 | 0.67 | - | 97.28 | 42.11 | 15.55 | 7.89 | **M**SG**S**LA**LM**Y**LN**S |
| NB-61 | 35.40 | 2.82 | 17.97 | - | 2.30 | 49.13 | 41.57 | 119.30 | TS**DS**L**ELM**Y**L**K**L** |
| NB-70 | 248.30 | 0.82 | 23.96 | - | 11.59 | 57.86 | 41.24 | 80.21 | TS**DS**L**ELM**Y**L**K**L** |

* Substitutions at amino acids 409/513/520/698/845/861/908/958/976/1076/1393**/**1450.
