# Supplementary figures and images for: Ex vivo susceptibilities of Plasmodium vivax isolates from the China-Myanmar border to antimalarial drugs and association with polymorphisms in Pvmdr1 and Pvcrt-o genes
Source: PLoS Negl Trop Dis. 2020 Jun 12;14(6):e0008255. doi: 10.1371/journal.pntd.0008255 (PMC7314094; doi:10.1371/journal.pntd.0008255)

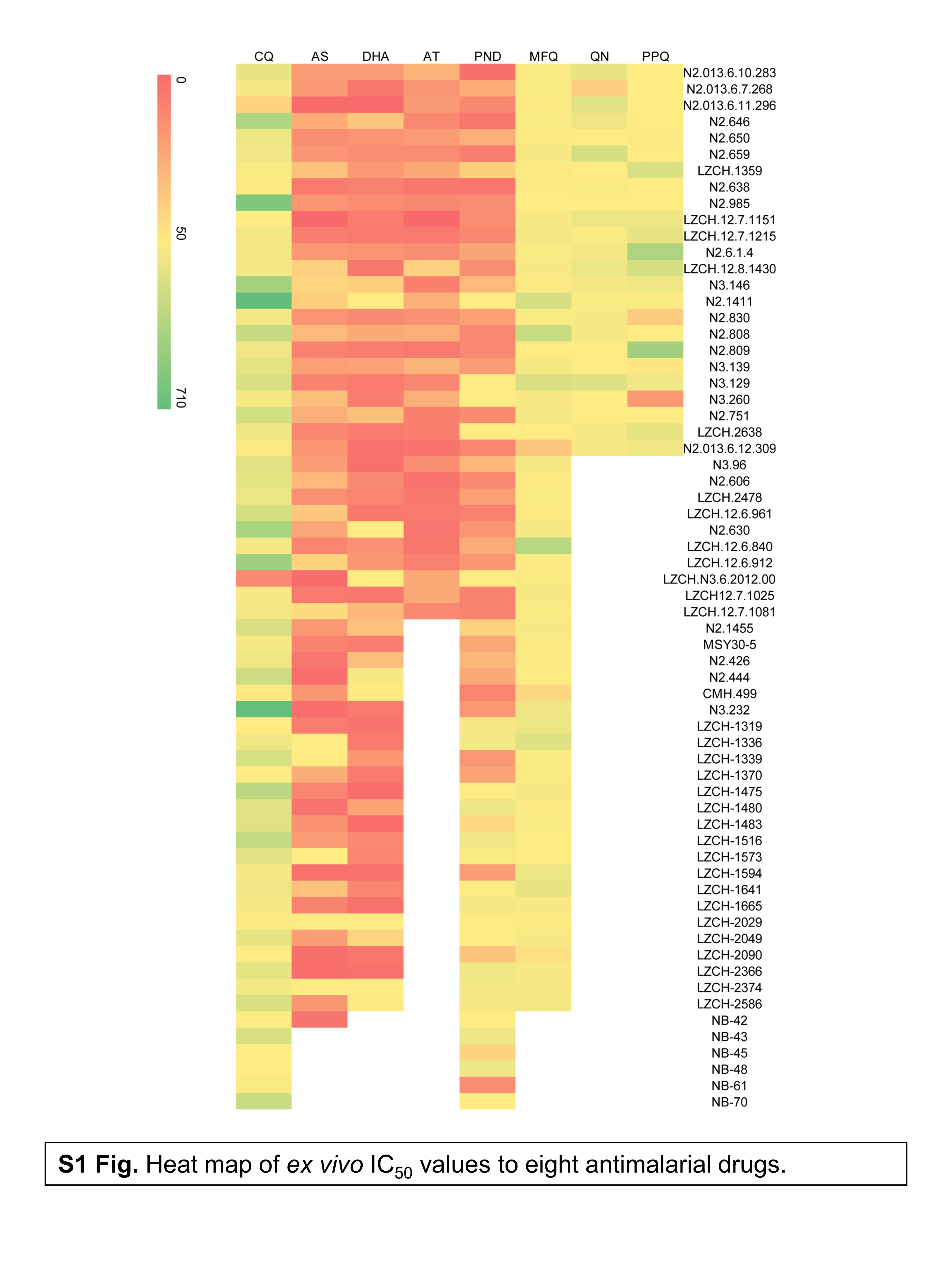

Supplement: S1 Fig — (TIF) [file pntd.0008255.s003.tif]

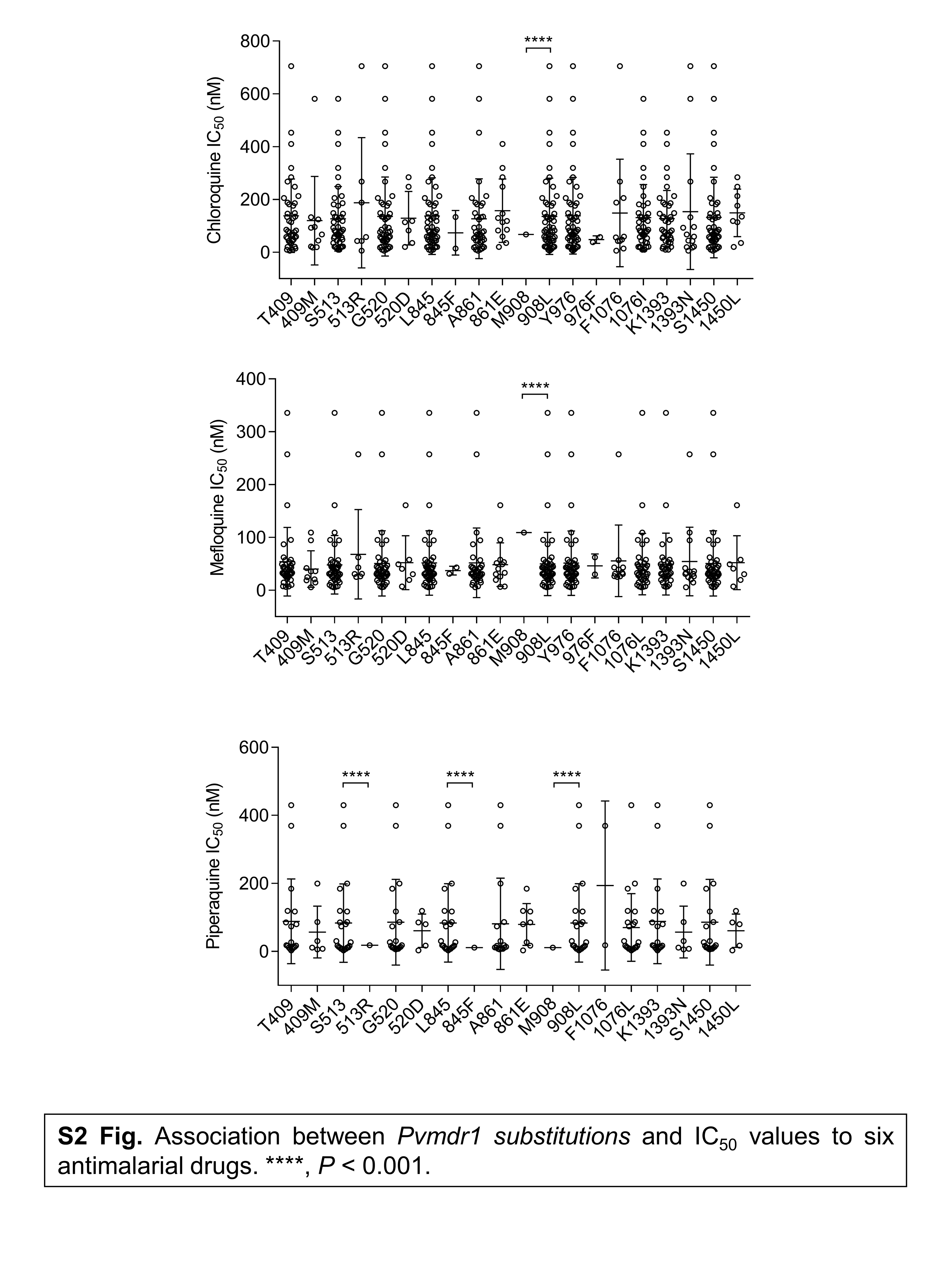

Supplement: S2 Fig — ****, P < 0.001. (TIF) [file pntd.0008255.s004.tif]

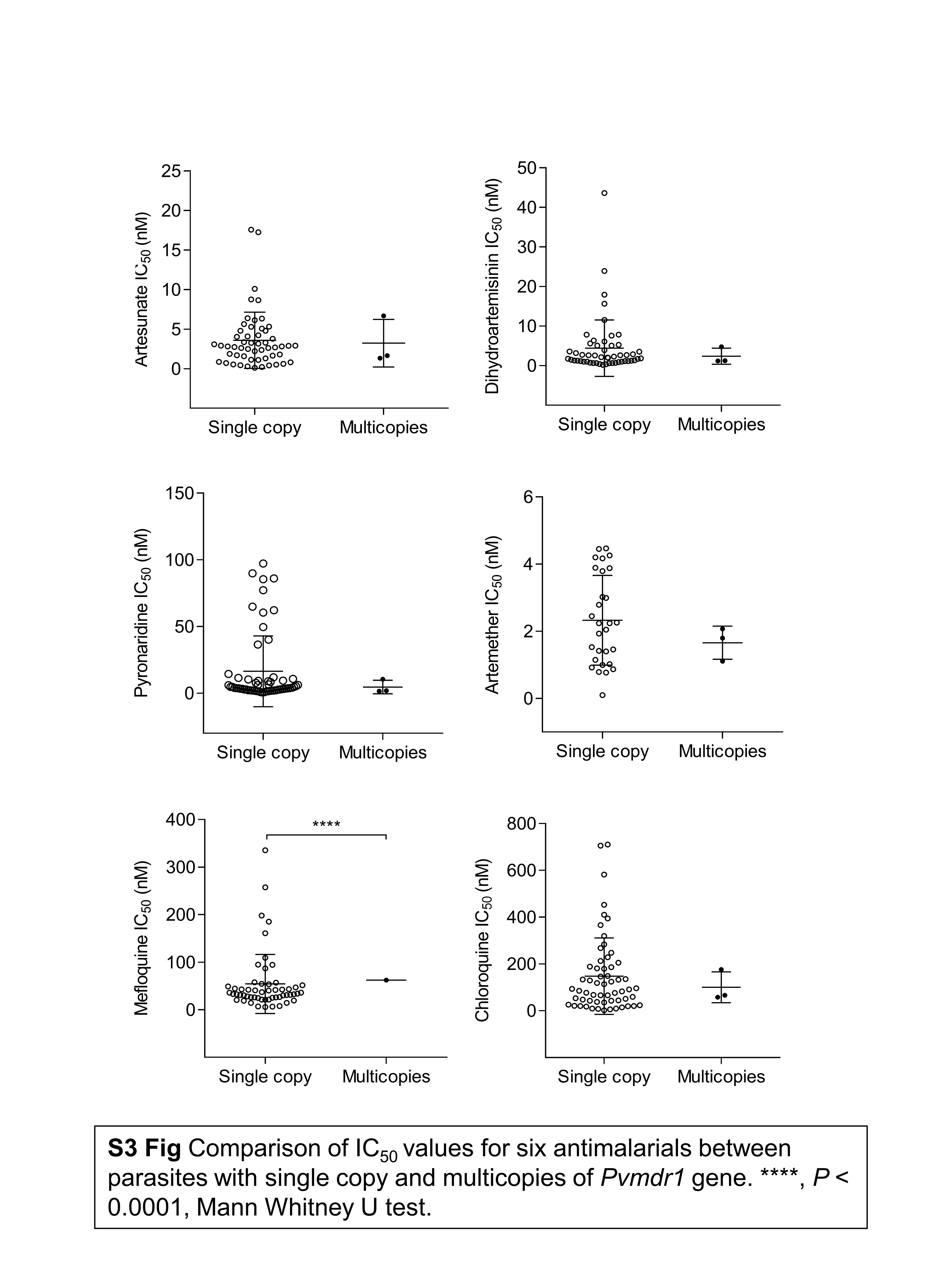

Supplement: S3 Fig — ****, P < 0.0001, Mann Whitney U test. (TIF) [file pntd.0008255.s005.tif]

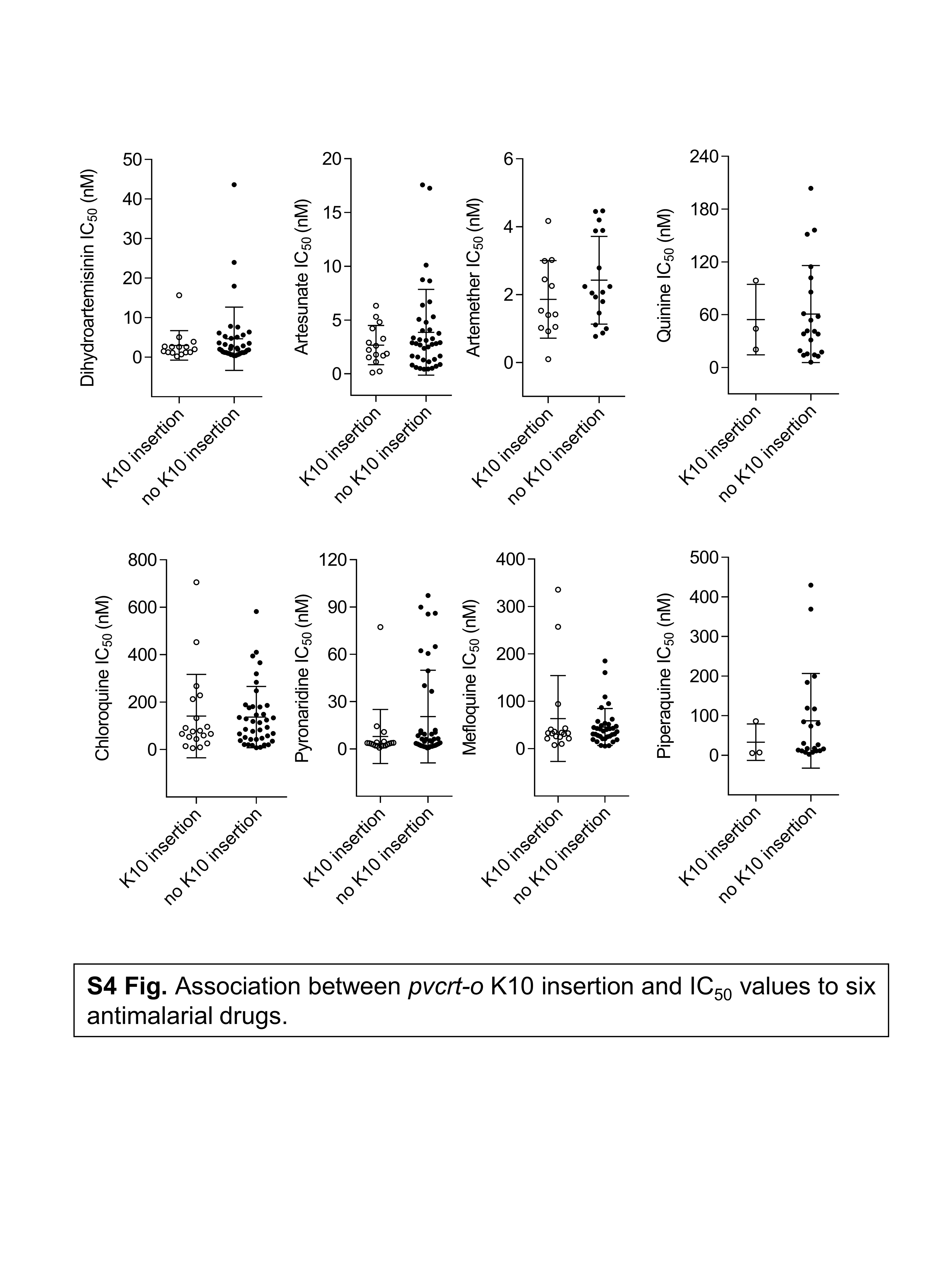

Supplement: S4 Fig — (TIF) [file pntd.0008255.s006.tif]
